# A Hint from America

**Published:** 1891-04-04

**Authors:** 


### A HINT FROM AMERICA.

Once more Miss Twining urges upon the public the necessity for Government providing women-attendants at police stations to attend upon women. Can there be anything more apparently needful than reform in this respect? We are very circumspect, we English, about some things, yet we are letting our friends across the ocean cut us out in this matter, for the Americans are passing a Bill to provide for the decent attendance on women under arrest. Good example is contagious sometimes; may it prove so on this occasion.

